# *Leishmania braziliensis* and *Leishmania amazonensis* amastigote extracts differ in their enhancement effect on *Leishmania* infection when injected intradermally

**DOI:** 10.1186/1756-0500-7-70

**Published:** 2014-02-01

**Authors:** Cintia F de Araújo, Virgínia MG Silva, Andre Cronemberger-Andrade, Luciana S Aragão-França, Viviane CJ Rocha, Priscila SL Santos, Lain Pontes-de-Carvalho

**Affiliations:** 1 Centro de Pesquisas Gonçalo Moniz. Fundação Oswaldo Cruz, Rua Waldemar Falcão 121, Salvador, Brazil; 2 State University of Southwest Bahia, Rua José Moreira Sobrinho, Jequié, Brazil

**Keywords:** *Leishmania amazonensis*, *Leishmania braziliensis*, Intradermal antigen, Infection enhancement, Leishmaniasis

## Abstract

**Background:**

It has been reported that repeated intravenous injections of a relatively large amount of *Leishmania amazonensis* amastigote extract (*La*E) in BALB/c mice exacerbates the infection of these mice by *Leishmania braziliensis*. The identification of the extract active principle(s) through physicochemical purification often involves dilution and losses of protein in the course of successive purification procedures. The large amount of the extract required to induce the phenomenon, therefore, hinders the carrying out of experiments aimed at identifying the active molecule(s) through extract purification. In the present work, a dose–response experiment was done to find out if smaller amounts of *La*E than that necessary to be used by the intravenous route would reproduce the phenomenon when injected by the intradermal route. In addition, it was also investigated whether a *Leishmania braziliensis* amastigote extract (*Lb*E) would exert the same effect and whether the effect would occur in C57BL/6 mice.

**Results:**

It was found that a single injection of either *La*E or *Lb*E containing 5 μg of protein was capable of enhancing the infection in BALB/c but not in C57BL/6 mice. In addition, it was observed that the largest tested doses of *Lb*E (containing 30 and 180 μg of protein) failed to enhance the infection by *L. braziliensis*, whereas all doses of *La*E enhanced equally that infection.

**Conclusions:**

Those results indicate the possible existence in *Lb*E, and not in *La*E, of molecules that interfere with the extract infection-enhancing activity when it is injected in large amounts, and that the inoculation of *Leishmania* extracts through the intravenous and intradermal routes potentiate the infection by L. braziliensis through the same mechanism.

## Background

Leishmaniases are zoonosis caused by different species of protozoa of the genus *Leishmania*. In Brazil, *Leishmania braziliensis* is the most prevalent etiologic agent in the northeast region and *Leishmania amazonensis* is the most widely distributed species [1,2]. Both species can cause American cutaneous leishmaniasis (ACL), which is in geographic expansion in Brazil, with the incidence increasing from 10.5/100,000 inhabitants in 1985 to 18.6/100,000 inhabitants in 2005. The disease has currently been reported in all regions of the country, but, with an average of 28,568 annual cases, the northeastern region, one of the poorest of the country, had the highest incidences between 1985 and 2005. In 2001, for example, the incidence in that region was 93.8 cases/100,000 inhabitants [3].

ACL has four distinct clinical manifestations: cutaneous, mucosal, disseminated cutaneous, and diffuse cutaneous. Cutaneous, mucosal, and disseminated leishmaniases are caused in Brazil mainly by *L. braziliensis*, while *L. amazonensis*, in addition to cutaneous leishmaniasis, causes, in a minority of cases, diffuse cutaneous leishmaniasis [4].

BALB/c mice are partially resistant to infection with *L. braziliensis*: they do not develop severe injuries and usually cure the infection with a mixed cellular immune response [5]. On the other hand, when infected by *Leishmania major* or *L. amazonensis*, either subcutaneously in the foot pad [6-8] or intradermally in the external ear [1,9], they develop a Th2-cell response and progressive skin lesions, which tend to be more necrotic in *L. major*-infected mice [10,11], and eventually die of the disease [11].

The susceptibility to the leishmanial infection depends not only on the *Leishmania* species, but also on the genetic background of the mouse. Thus, C57BL/6, CBA and BALB/c mice are resistant to *L. braziliensis* and susceptible to *L. amazonensis*[12,13]. On the other hand, contrasting with BALB/c mice, C57BL/6 and CBA mice are resistant to *L. major*[10].

The outcome of the infection of BALB/c mice by *L. braziliensis*, however, can be completely changed by treating them with four biweekly intravenous injections of *L. amazonensis* amastigote extract containing 200 μg of protein and starting one week before the infection: the animals thus treated become fully susceptible to the disease. On the other hand, this phenomenon did not take place when a *L. braziliensis* amastigote extract or a *L. amazonensis* promastigote extract was used instead of the *L. amazonensis* amastigote extract [8], or when lower amounts of *L. amazonensis* amastigote extract were used [Silva VMG, unpublished].

The identification of the extract active principle by its purification to homogeneity very often entails its dilution or partial loss during two or more consecutive purification steps. For this reason, it would be important to find out whether the infection-enhancing phenomenon could be observed with lower amounts of the extract than that required by the intravenous route if it was injected by another route. The aim of the present study was to investigate whether lower doses of the *L. amazonensis* amastigote extract (*La*E) could modulate the *L. braziliensis* infection in BALB/c and C57BL/6 mice when injected by the intradermal route and whether this effect could be observed with *L. braziliensis* amastigote extract (*Lb*E). It was found that a single injection of either *La*E or *Lb*E in a dose two orders of magnitude lower than that needed intravenously [8] induced the phenomenon in BALB/c mice. In addition, the highest doses of *Lb*E failed to enhance the infection in BALB/c mice, and none of the tested doses enhanced the infection in C57BL/6 mice.

## Results

### Effect of the intradermal injection of *La*E on *L. braziliensis* infection in BALB/c mice

In previous experiments carried out by our research group, an amount of *La*E containing at least 200 μg of protein was required to enhance a *L. braziliensis* cutaneous infection in BALB/c mice ( [8]; Silva et al., unpublished results). The requirement to inject this relatively large amount of extract in each mouse, in experiments with 6 to 8 mice per group, practically precludes the purification of the active principle from research laboratory’s parasite batches by means of biophysical techniques, such as electrophoresis and liquid chromatography. One of the aims of the present work, therefore, was to find out whether smaller amounts of extract, when injected by another route, would also mediate the phenomenon.

**Figure 1:**
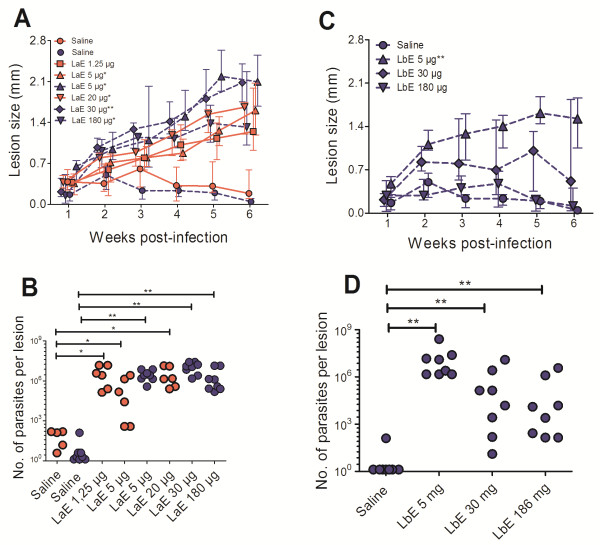
**Effect of intradermal injection of *L. amazonensis *(LaE) and *L. braziliensis *(LbE) extracts in the infection of BALB/c mice by *L. braziliensis*. A** and **C**, lesion sizes in groups of five to eight BALB/c mice injected with saline and different doses of LaE **(A)** or LbE **(C). B** and **D**, number of parasites per lesion as estimated by limiting dilution, at the sixth week after infection, of the same mice whose lesion sizes are depicted in A and C, respectively. The animals were treated with a single intradermal injection of saline or of the doses of extracts indicated above the graphs in A and C or below the X axis in B and D. One week later they were infected with 10^7^ *L. braziliensis* promastigotes in the stationary phase of growth. The lines in A and C join the median values of the results from each group in each week, and the vertical bars represent the interquartile interval. Each symbol in B and D represents the result obtained from a single animal. The results of two independent experiments are shown. Symbols and lines corresponding to the results from one experiment are dark blue and the lines are broken; from the other experiment the symbols and lines are red and the lines are continuous. Comparisons between the groups of extract-injected and the group of saline-injected mice were performed by Mann–Whitney *U* test [level of significance when comparing lesion sizes **(A and C)** = 0.0056; when comparing lesion parasitism **(B and D)** = 0.0167]. *P < 0.005; **P < 0.001.

In a first experiment, doses of extract containing 1.25, 5, and 20 μg of protein were used. It was found that a single intradermal injection of *La*E, containing amounts of protein as little as 1.25 μg, led to significant increases in the sizes of the lesions caused by *L. braziliensis* (Figure 1A) and in tissue parasitism (Figure 1B). The smallest dose tested—1.25 μg—led to significant increases in lesion parasitism (Figure 1B), but not in lesion sizes (Figure 1A).

### Dose-responses of the effect of *Lb*E and *La*E intradermal injections in *L. braziliensis*-infected BALB/c mice

We next decided to find out how comparable were the results that could be produced by administering the extract through the intradermal route with those previously published by Silvia and collaborators, who injected the extract by the intravenous route [8]. Because, when injected intravenously, the *Lb*E failed to enhance an *L. braziliensis* infection, we investigated whether the same would occur when the *Lb*E was injected intradermally. In this second experiment, doses of extracts containing 5, 30 and 180 μg of protein were used because one of them (the dose of 5 μg of protein) fell within the range of *La*E doses shown in the present work to enhance the infection (doses containing 1.25 to 20 μg of protein), and another of them was 9 folds higher (the dose of 180 μg of protein) than the highest amount (20 μg) of *La*E that we had tested so far.

The injection of *Lb*E containing 5 μg of protein significantly increased the size of the lesions caused by *L. braziliensis* in BALB/c mice (Figure 1C). This did not occur when the dose was augmented to 30 and 180 μg of protein: the sizes of lesions of mice injected with these latter doses of *Lb*E did not differ significantly from those of mice injected only with saline (Figure 1C). These findings contrasted markedly with what was observed when the mice were injected with the same doses of *La*E: all these doses of *La*E (containing 5, 30 and 180 of protein) caused a significant increase in lesion sizes (Figure 1A).

As expected from the results described above, the reduction in lesion size with the increasing doses of *Lb*E was statistically significant (Table 1). Thus, the group of mice injected with the 5 μg of protein *Lb*E dose significantly differ from the groups of mice injected with the 30 μg dose (as determined by Fisher’s exact probability test) and with the 180 μg dose (as determined by Fisher’s exact probability and Mann–Whitney U tests; Table 1).

**Table 1:** **Statistical significance of differences in the potentiating effect of** ***L. amazonensis *** **(*La*** **E) and *L. braziliensis*** **(*Lb*** **E) extracts on *L. braziliensis *** **infection, and between different doses of the same extract**

**Tested extracts in compared groups**	**Measured outcome**	**Protein amount in extracts (μg)**	**P**	**Significance level**	**Test**
		5	0.1074	0.0056	Mann–Whitney U
			0.5000		Fisher’s exact probability
	Lesion size	30	0.0079		Mann–Whitney U
			0.0035*		Fisher’s exact probability
		180	**0.0046**		Mann–Whitney U
			**0.0051**		Fisher’s exact probability
*La*E, *Lb*E		5	0.1469	0.0167	Mann–Whitney U
			0.5000		Fisher’s exact probability
	Lesion parasitism	30	**0.0043**		Mann–Whitney U
			0.0385		Fisher’s exact probability
		180	**0.0136**		Mann–Whitney U
			**0.0035**		Fisher’s exact probability
		5, 30	1.0000	0.0083	Mann–Whitney U
			1.0000		Fisher’s exact probability
	Lesion size	5, 180	0.2263		Mann–Whitney U
			1.0000		Fisher’s exact probability
*La*E		5, 30	**0.0177**	0.0250	Mann–Whitney U
			1.0000		Fisher’s exact probability
	Lesion parasitism	5, 180	0.1902		Mann–Whitney U
			1.0000		Fisher’s exact probability
		5, 30	0.0658	0.0083	Mann–Whitney U
			**0.0070**		Fisher’s exact probability
	Lesion size	5, 180	**0.0019**		Mann–Whitney U
			**0.0014**		Fisher’s exact probability
*Lb*E		5, 30	**0.0155**	0.0250	Mann–Whitney U
			0.0769		Fisher’s exact probability
	Lesion parasitism	5, 180	**0.0039**		Mann–Whitney U
			**0.0070**		Fisher’s exact probability

*Boldface values are statistically significant.

Contrasting with the described above for the *Lb*E*,* no statistically significant difference in lesion sizes was observed between the groups of mice injected with 5 and 180 μg doses of *La*E (Figure 1A and Table 1). The lesion sizes in the group of mice injected with the 30 μg dose of *La*E significantly differ from those of the mice injected with the 5 μg dose of that extract, as shown by Mann–Whitney *U* test (Table 1). However, this dose led to larger lesions than the 5 μg dose (Figure 1A), and not to the opposite, as happened with *Lb*E.

All doses of *Lb*E increased tissue parasitism (Figure 1D). However, the proportion of mice in which the injection of the lowest amount of *Lb*E (containing 5 μg of protein) led to more than 20,000 parasites per lesion (8 out of 8 mice) was significantly larger (p = 0.007, Fisher’s exact probability test, significance level = 0.025; Table 1) than the proportion of the mice in which the highest amount (with 180 μg of protein) led to more than 20,000 parasites per lesion (2 out of 8 mice; Figure 1D). In fact, 8 out of 8 mice that received *Lb*E containing 5 μg of protein had more than 1,500,000 parasites per lesion (Figure 1D). On the other hand, all doses of *La*E led to similar increases in tissue parasitism (Figure 1B).

In accordance with the data described above, *Lb*E was less active than *La*E in terms of increasing lesion sizes in the dose of 30 μg (as shown by Fisher’s exact probability test; Table 1) and in the dose of 180 μg (in accordance both with Fisher’s exact probability and with Mann–Whitney U tests; Table 1). In terms of enhancing lesion parasitism, again *Lb*E was less active than *La*E in the dose of 30 μg (as shown by Mann–Whitney *U* test; Table 1) and in the dose of 180 μg (in accordance with both Fisher’s exact probability and Mann–Whitney U tests; Table 1). No statistically significant differences between *Lb*E and *La*E was observed when they were administered in the dose of 5 μg of protein (Table 1).

### Effect of the intradermal injection of *La*E or *Lb*E on *L. braziliensis* infection in C57BL/6 mice

When injected intravenously, *La*E failed to potentiate the infection by *L. braziliensis* in C57BL/6 mice [8]. In order to further compare the results that could be obtained through injection of the extract through the intradermal route with those previously described with injection by intravenous route, C57BL/6 mice were injected by the intradermal route with a dose of *La*E and of *Lb*E containing 5 μg of protein, an amount that clearly enhanced the infection in BALB/c mice (Figures 1A and 1C). As a positive control of the infection-enhancing activity of the *La*E, this extract was also injected into BALB/c mice.

**Figure 2:**
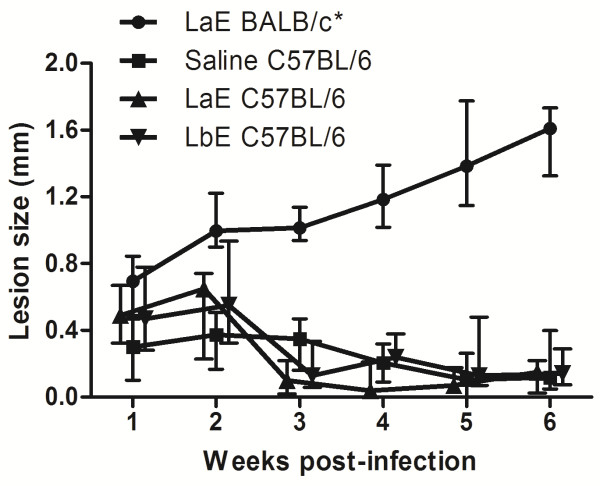
**Lesions sizes in *L. braziliensis*-infected C57BL/6 mice in which *L. amazonensis *and *L. braziliensis *extracts had been injected.** The animals (n = 4–6 per group) were treated with intradermal injections of the vehicle (Saline C57BL/6) or of *L. amazonensis* (LaE BALB/c and LaE C57BL/6) or *L. braziliensis* (LbE C57BL/6) extracts containing 5 μg of protein, one week before being infected with 10^7^ *L. braziliensis* promastigotes in the stationary phase of growth. The lines in A and C join the median values of the results from each group in each week, and the vertical bars represent the interquartile interval. Comparisons between the groups of extract-injected and the group of saline-injected mice were performed by Mann–Whitney *U* test (level of significance = 0.0056). *P < 0.001.

The infection of the C57BL/6 mice by *L. braziliensis* led to quickly resolving lesions (as judged by their sizes), with less than 1 mm of thickening of the mouse foot pads, that started to subside after the second week but were still perceptible in most mice, after six weeks, as less than 0.2 mm foot pad enlargement (Figure 2 and data not shown).

Neither the intradermal injection of *La*E nor of *Lb*E exacerbated the cutaneous disease caused by *L. braziliensis* in those mice (Figure 2).

## Discussion

As has been shown for the intravenous injection, the intradermal injection of *La*E in BALB/c mice potentiated the infection by *L. braziliensis.* However, when administered by the intradermal route, a dose of *La*E two orders of magnitude lower (1.25 μg) than the dose that was able to promote the infection by the intravenous route (200 μg; [8]) was enough to produce the effect. Differences in *Leishmania* infection outcomes depending on the route of administration of the antigen have been observed [4,14,15]. However, comparisons between immunizations with *Leishmania* antigens by the intravenous and the intradermal routes have not been so far reported.

The effect on the size of the lesions caused by the 1.25 μg of protein dose was very apparent. However, the effect, at this low dose of the extract, was not statistically significant (p = 0.0102, Mann–Whitney *U* test), perhaps due to the fact that three different comparisons had been made (namely 1.25 μg vs saline, 5 μg vs saline, and 20 μg vs saline) in three different occasions (in which differences in lesion sizes could be expected: the 4th, the 5th, and the 6th weeks). These multiple comparisons reduced the level of significance to 0.0056. The conspicuousness of the extract effect in this low dose, however, increases the feasibility of the physicochemical purification of its active principle from amounts of extract easily obtainable in a research facility.

When injected intravenously, the enhancing effect on the infection of *Leishmania* extracts depends on the presence of IL-4 [8]. The mechanism of this enhancement when the antigen is injected into the intradermal space, however, has still to be clarified. Preliminary experiments showed no differences in the production of either IL-4 or IL-10 by anti-CD3 - stimulated cells of lesion-draining lymph nodes (unpublished). The possibility that the effect could be mediated by TGF-β-producing Treg cells is open to investigation. Supporting this hypothesis, it has been reported that TGF-β exacerbates the infection of BALB/c mice by *L. amazonensis*[16,17]. If the most likely underlying mechanism, i. e., the biasing of the BALB/c mouse immune response towards an infection-permissive one by antigens in the *La*E is true, the C57BL/6 immune system would respond to these antigens in a different way, as the *La*E did not increase lesion sizes in C57BL/6 mice.

It was also demonstrated in the present work that the injection of *LbE* by the intradermal route potentiates the infection by *L. braziliensis*. However, contrasting to what was observed with *La*E, a statistically significant effect was only seen with a lower dose of the extract and not with the larger doses. The infection-enhancing activity of the largest doses of *Lb*E was indeed significantly lower than the activivities of the same doses of *La*E. This finding is consistent with that reported by Silva and collaborators [8], who found that *Lb*E, when repeatedly administered by the intravenous route in a relatively high dose, did not exacerbate the infection of BALB/c mice by *L. braziliensis*. This dose-dependent difference between *La*E and *Lb*E in affecting the course of the disease could be ascribed to the coexistence in *Lb*E of molecules that could induce protective immune responses and of others that could induce a permissive immune response, which would have different dose (of the extract)-response curves. Thus, antigens that could induce a protective immune response would be recognized by the immune system only when high amounts of *Lb*E was introduced in the organism (and identical or similar antigens would not be present or be present in an insufficient amount in the *La*E), whereas in low amounts of *Lb*E other antigens, which could be present in higher concentrations in the extract, would induce a susceptibility-associated immune response. This hypothesis is amenable to investigation, e.g. through experiments using *Lb*E fractions and/or by testing the effect of mixtures of low amounts of *La*E with high amounts of *Lb*E.

These results indicate, therefore, that the two *Leishmania* species may differ in terms of the presence, or of the relative amounts, of antigens against which the BALB/c immune system would mount a protective immune response. Whether this difference would account for the fact that a minority of human beings with cutaneous lesions caused by *L. amazonensis* develops a progressive disease, a phenomenon that does not occur when the cutaneous lesions are caused by *L. braziliensis*, is open to speculation. The progressive disease would be accounted by *L. amazonensis* parasites producing mainly infection-promoting molecules, whose recognition as such would also depend on the genetic background of the host. It is tempting to make a parallel between the fact that only the BALB/c, and not the C57BL/6 mice, had their disease worsened by *Leishmania* extracts and the fact that only a minority of human beings with clinically manifested *L. amazonensis* infection develop the diffuse, progressive disease.

Evidence for the existence of different immunomodulatory *Leishmania* molecules that might potentiate the infection have been reported [17-22]. A preliminary fractionation of the *La*E by polyacrylamide gel electrophoresis followed by electroelution of proteins from gel fragments and analysis of the eluted protein fractions by mass spectrometry led to the conclusion that at least three different *Leishmania* proteins are able to enhance the *Leishmania* infection in BALB/c mice when injected intradermally. One of the proteins present in a fraction with infection-enhancing activity was the *Leishmania* homologue of receptors for activated C kinase (LACK), which has been described as having immunomodulatory activity [19]. Two other fractions with *Leishmania* infection-enhancing activity contained proteins to which no immunomodulatory activity has been attributed so far (unpublished).

Different *L. braziliensis* isolates may cause different intensities of cutaneous disease and lesions with distinct histopatological patterns [5,23]. The isolate we used causes a self-healing cutaneous disease when inoculated in the foot pad of BALB/c mice, with lesions smaller than 1 mm that usually subside after two or three weeks and are bearly measurable after six weeks. However, about one in every three or four mice develops a larger lesion, that also subsides after six weeks (unpublished observations). It would not be surprising if extracts prepared from different *L. braziliensis* isolates would differ in their infection-enhancing activity.

Moreover, the fact that a high amount of *Lb*E is less active in terms of inducing disease exacerbation than lower amounts of the same extract and than the same high amount of *La*E can be used as evidence against the hypothesis that the simple introduction of any *Leishmania* antigen in the intradermal compartment would aggravate the disease.

## Conclusions

In conclusion, it was demonstrated in the present work that a few micrograms of *La*E or *Lb*E, when injected intradermally, are capable of aggravating the cutaneous disease caused by *L. braziliensis* in BALB/c mice, and that this phenomenon is not seen with larger amounts of *Lb*E.

As has been reported previously, *La*E failed to enhance the infection in C57BL/6 mice when injected intravenously [8]. As shown in the present work, this failure also occurs when it is injected intradermally. In addition, the *Lb*E was less active in promoting the infection than the *La*E when injected in relatively high doses by either one of the two routes ( [8] and the present work). Taken together, these findings indicate that the same mechanism is underlying the observed phenomena when the extract is injected intravenously or intradermally.

## Methods

### Mice and ethical considerations

Specific-pathogen-free, 8 to 12 week-old BALB/c and C57BL/6 mice were maintained at the animal facilities of the Gonçalo Moniz Research Center, Oswaldo Cruz Foundation, Salvador, Brazil, and were provided with rodent diet and water *ad libitum*. All procedures were approved and conducted according to the institutional Committee for Animal Care and Utilization.

### Parasites and parasite extracts

*L. amazonensis (*MHOM/Br87/Ba125) and *L. braziliensis (*MHOM/Br/3456) strains were used. Their infectivities were maintained by regular inoculations of promastigotes into susceptible BALB/c mice and golden hamsters, respectively.

Promastigotes, derived from tissue amastigotes, were cultured at 23°C in Schneider’s medium (Sigma Chemical Co., St. Louis, MO, USA), pH 7.2, supplemented with 50 μg/mL of gentamicin and 10% heat-inactivated fetal bovine serum (FBS; Gibco Laboratories, Grand Island, NY) for *L. amazonensis* or 20% FBS for *L. braziliensis*.

*L. amazonensis* and *L. braziliensis* axenic amastigotes were obtained by the differentiation of promastigotes in axenic cultures, as described elsewhere [24]. The amastigotes were washed three times in ice cold sterile saline, resuspended in saline, and lysed by exposure to ultrasound (10 1-minute, 300-W pulses, with 30-second intervals in between, on ice; Sonifier Cell Disruptor; Branson Sonic Power Company, Danbury, CT, USA). The lysates were centrifuged at 16,000 *g* for 10 min at 4°C, the supernatants were filtered on membranes with 0.22-μm diameter pores (Millipore, São Paulo, Brazil) and immediately aliquoted and stored at -70°C.

### Infection and treatment of animals, determination of lesion size and experimental design

*L. braziliensis* promastigotes (10^7^), obtained from stationary-phase cultures, were subcutaneously inoculated into one of the hind footpads of BALB/c or C57BL/6 mice [16,24] one week after their being injected in the dermis of the dorsal region, with 50 μL of *La*E or *LbE* containing the amounts of protein that are indicated in the Figures. The hind pad thicknesses were weekly monitored with a digital caliper, until the sixth week post-infection and the lesion sizes estimated by subtracting the thickness of the uninfected pad from the thickness of the infected pad. Parasite loads in the footpads were estimated by limiting dilution [25] at the end of the experiments, as described below.

### Quantification of tissue parasitism

Briefly, the infected pads were macerated in Schneider’s medium and centrifuged at 50 *g* for 10 min, at 4°C. The supernatants were recentrifuged at 1,540 *g* for 10 min at 4°C, and the pellets were resuspended in Schneider’s medium supplemented with 50 μg/mL gentamicin and 20% FBS. The suspension was serially diluted in 2-fold dilutions and distributed in triplicate in 96-well culture plates. The number of viable parasites in each footpad was determined from the reciprocal of the highest dilution at which promastigotes could be detected after 7 days at 23°C and was expressed as the number of parasites per lesion.

### Statistical analyses

The type of data distribution was determined by the Shapiro-Wilk test. Because the distribution was found to be non-Gaussian, non-parametric tests were used.

The statistical significance of differences in lesion size and parasitism between pairs of groups of mice subjected to different treatments were estimated by the Mann–Whitney *U* test. As explained below, the number of compared pairs in each experiment was taken into account to calculate the significance level.

To additionally assess the statistical significance of differences in lesion sizes, and in intensity of lesion parasitism, between the groups of mice that had received 5 and 30 μg of extract, between the groups of mice that had received 5 and 180 μg of extract, and between the groups of mice that had received *Lb*E and *La*E, these groups of mice were stratified into two pairs of categories: mice with lesions larger than 1 mm and mice with lesions smaller than 1 mm; mice with lesions with more than 20,000 parasites and mice with lesions with less than 20,000 parasites. Two × 2 contingency tables associating the numbers of mice falling within the categories with one or another of two experimental protocols (the protocol in which the mice received 5 μg of extract and the protocol in which they received 30 μg of the same extract; the protocol in which the mice received 5 μg of extract and the protocol in which they received 180 μg of the same extract; and the protocol in which the mice received a given amount of *La*E and the protocol in which they received the same amount of *Lb*E) were constructed. Fisher’s exact probability test was used to assess whether the frequency distributions of data within these tables were due to chance, since that test was designed for contingency tables with a small sample size, like the one in the present study.

Results were considered significant when the value of *P* was ≤0.05 divided by the number of pairs of compared datum sets in the statistical test.

## Abbreviations

ACL: American cutaneous leishmaniasis; FBS: Fetal bovine serum; IL-: Interleukin; LaE: *Leishmania amazonensis* amastigote extract; LbE: *Leishmania braziliensis* amastigote extract; TGF-β: Transforming growth factor beta; Th2: T helper type 2.

## Competing interests

The authors declare that they have no competing interests.

## Authors’ contributions

CFde-A carried out most of the experiments and wrote a first draft of the manuscript; VMGS helped in designing the experiments; ACA, LSA-F, VCJR, and PSLS carried out some of the experimental work; LP-de-C devised and designed the experiments and wrote the final version of the manuscript. All authors read and approved the final manuscript.

## References

[B1] Belkaid Y Kamhawi S Modi G Valenzuela J Noben-Trauth N Rowron E Ribeiro J Sacks DL Development of a natural model of cutaneous leishmaniasis: powerful effects of vector saliva and saliva pre exposure on the long-term outcome of *Leishmania major* infection in the mouse ear dermis J Exp Med 1998 188 1941 1953 10.1084/jem.188.10.1941 9815271 PMC2212417

[B2] De Oliveira C Barral-Netto M O modelo experimental nas infecções causadas por *L. amazonensis e L. braziliensis* Gaz Med Bahia 2005 75 35 45

[B3] Departamento de Vigilância Epidemiológica, Secretaria de Vigilância em Saúde, Ministério da Saúde Manual de controle da leishmaniose tegumentar americana 2007 Brasília: MS Editora

[B4] Carvalho PL Passos TS Jesus RA Immunopathogenesis of tegumentary leishmaniasis Gaz Med Bahia 2005 1 57 65

[B5] Indiani de Oliveira C Teixeira MJ Teixeira CR Ramos de Jesus J Bomura Rosato A Santa da Silva J Brodskyn C Barral-Netto M Barral A *Leishmania braziliensis* isolates differing at the genome level display distinctive features in BALB/c mice Microbes Infect 2004 6 977 984 10.1016/j.micinf.2004.05.009 15345228

[B6] Scott PA Farrell JP Experimental cutaneous leishmaniasis: disseminated leishmaniasis in genetically susceptible and resistant mice Am J Trop Med Hyg 1982 31 230 238 6462064 10.4269/ajtmh.1982.31.230

[B7] Granfell RFQ Marques-da-Silva EA Souza-testasicca MC Coelho EA Fernandes AP Afonso LC Rezende SA Antigenic extracts of *Leishmania braziliensis* and *Leishmania amazonensis* associated with saponin partially protects BALB/c mice against *Leishmania chagasi* infection by suppressing IL-10 and IL-4 production Mem Inst Oswaldo Cruz 2010 105 818 822 10.1590/S0074-02762010000600015 20944999

[B8] Silva VMG Larangeira DF Oliveira PRS Sampaio RB Suzart P Nihei J Teixeira MCA Mengel JO Dos Santos WLC Pontes-de-Carvalho L Biointervention Student Group Enhancement of experimental cutaneous leishmaniasis by *leishmania* molecules with serine protease activity. I. Requirement of IL-4 Infect Immun 2011 79 1236 1243 10.1128/IAI.00309-10 21173308 PMC3067519

[B9] Courret N Lang T Milon G Antoine JC Intradermal inoculations of low doses of *Leishmania major* and *Leishmania amazonensis* metacyclic promastigotes induce different immunoparasitic processes and status of protection in BALB/c mice Int J Parasitol 2003 33 1373 1380 10.1016/S0020-7519(03)00179-6 14527520

[B10] Silva-Almeida M Carvalho POL Abreu-Silva LA Souza FSC Hardoim JD Extracellular matrix in experimental *Leishmania amazonensis* infection in susceptible and resistant mice Vet Res 2012 43 1 9 10.1186/1297-9716-43-1 22316002 PMC3395857

[B11] Weiss R Scheiblhofer S Thalhamer J Bickert T Richardt U Fleischer B Ritter U Epidermal inoculation of *Leishmania*-antigen by gold bombardment results in a chronic form of leishmaniasis Vaccine 2007 25 25 33 10.1016/j.vaccine.2006.07.044 17064826

[B12] Childs GE Lightner LK Mckinney L Groves MG Price EE Hendricks LD Inbred mice as model hosts for cutaneous leishmaniasis. I. Resistance and susceptibility to infection with *Leishmania braziliensis*, *L. mexicana*, and *L. aethiopica* Ann Trop Med Parasitol 1984 78 25 34 6721612 10.1080/00034983.1984.11811769

[B13] Felizardo TC Gaspar-Elsas MI Lima GM Abrahamsohn IA Lack of signaling by IL-4 or by IL-4/IL-13 has more attenuating effects on *Leishmania amazonensis* dorsal skin- than on footpad-infected mice Exp Parasitol 2012 130 48 57 10.1016/j.exppara.2011.09.015 22019418

[B14] Aebischer T Morris L Handman E Intravenous injection of irradiated *Leishmania* major into susceptible BALB/c mice: immunization or protective tolerance Int Immunol 1994 10 1535 1543 7826944 10.1093/intimm/6.10.1535

[B15] Bhowmick S Mazumdar T Ali N Vaccination route that induces transforming growth factor β production fails to elicit protective immunity against *Leishmania donovani* infection Infect Immun 2009 77 1514 1523 10.1128/IAI.01739-07 19168736 PMC2663136

[B16] Sacks D Noben-Trauth N The immunology of susceptibility and resistance to Leishmania major in mice Nat Rev Immunol 2002 2 845 858 10.1038/nri933 12415308

[B17] Pinheiro RO Pinto EF Lopes JRC Guedes HLM Fertanes FR Rossi-Bergmann B TGF-β-associated enhanced susceptibility to leishmaniasis following intramuscular vaccination of mice with *Leishmania amazonensis* antigens Microbes Infect 2005 7 1317 1323 10.1016/j.micinf.2005.04.016 16027022

[B18] de Assis RR Ibraim IC Nogueira PM Soares RP Turco SJ Glycoconjugates in New World species of Leishmania: polymorphisms in lipophosphoglycan and glycoinositolphospholipids and interaction with hosts Biochim Biophys Acta 1820 2012 1354 1365 10.1016/j.bbagen.2011.11.001 22093608

[B19] Julia V Rassoulzadegan M Glaichenhaus N Resistance to *Leishmania major* induced by tolerance to a single antigen Science 1996 274 421 423 10.1126/science.274.5286.421 8832890

[B20] Lacerda ID Cysne-Finkelstein L Numed PM de-Luca PM Genestra SM Leon PLL Pinho BM Lima ML Matos SCD Medeiros AM MENDONçA SFC Kinetoplastid membrane protein-11 exacerbates infection with Leishmania amazonensis in murine macrophages Memórias Instituto Oswaldo Cruz 2012 107 238 245 10.1590/S0074-02762012000200014 22415264

[B21] Yao C Donelson JE Wilson ME The major surface protease (MSP or GP63) of Leishmania sp. Biosynthesis, regulation of expression, and function Mol Biochem Parasitol 2003 132 1 16 10.1016/S0166-6851(03)00211-1 14563532

[B22] Wanderlei MLJ Costa FJ Borges MV Barcinski M Subversion of Immunity by Leishmania amazonensis Parasites: Possible role of phosphatidylserine as a main regulator J Parasitol Res 2012 2012 981186 10.1155/2012/981686 PMC3306939 22518276

[B23] Pereira CG Silva AL de Castilhos P Mastrantonio EC Souza RA Romão RP Rezende RJ Pena JD Beletti ME Souza MA Different isolates from *Leishmania braziliensis* complex induce distinct histopathological features in a murine model of infection Vet Parasitol 2009 165 231 240 10.1016/j.vetpar.2009.07.019 19656631

[B24] Teixeira MC De Jesus SR Sampaio RB Pontes De Carvalho LC Dos Santos WC A simple and reproducible method to obtain large numbers of axenic amastiotes of different *Leishmania* species Parasitology 2002 88 963 968 10.1007/s00436-002-0695-3 12375160

[B25] Lima HC Bleyenberg JA Titus RG A simple method for quantifying *Leishmania* in tissues of infected animals Parasitol Today 1997 13 80 82 10.1016/S0169-4758(96)40010-2 15275128

